# Exploration of the effects of Coriolis force and thermal radiation on water-based hybrid nanofluid flow over an exponentially stretching plate

**DOI:** 10.1038/s41598-022-21799-9

**Published:** 2022-12-16

**Authors:** A. S. Oke, B. C. Prasannakumara, W. N. Mutuku, R. J. Punith Gowda, B. A. Juma, R. Naveen Kumar, O. I. Bada

**Affiliations:** 1 Department of Mathematical Sciences, Adekunle Ajasin University, Akungba Akoko, Nigeria; 2 Department of Mathematics and Actuarial Science, Kenyatta University, Nairobi, Kenya; 3 Department of Studies and Research in Mathematics, Davangere University, Davangere, India; 4 Department of Mathematics, University of Benin, Benin City, Nigeria

**Keywords:** Applied mathematics, Mechanical engineering

## Abstract

Hybrid nanofluids’ enhanced thermophysical properties make them applicable in a plethora of mechanical and engineering applications requiring augmented heat transfer. The present study focuses on a three-dimensional Copper-Aluminium Oxide $\left( Cu\text{- }Al_{2}O_{3}\right)$-water based hybrid nanofluid flow within the boundary layer with heat transfer over a rotating exponentially stretching plate, subjected to an inclined magnetic field. The sheet rotates at an angular velocity $\Omega$ and the angle of inclination of the magnetic field is $\gamma$. Employing a set of appropriate similarity transformation reduces the governing PDEs to ODEs. The resulting ODEs are solved with the finite difference code with Shooting Technique. Primary velocity increases at large rotation but the secondary velocity reduces as the rotation increases. In addition, the magnetic field is found to oppose the flow and thereby causing a reduction in both the primary and secondary velocities. Increasing the volume fraction reduces the skin friction coefficient and enhances the heat transfer rate.

## Introduction

The field of nanotechnology has captivated the interest of researchers in the recent decades. Nanoliquids compose of some carrier liquids such as water, with some solid nanoparticles (particles less than 100 nm in diameter). Applications of the nanoliquids are in power-plants, nuclear reactor cooling, aircraft, and micro-reactors. Firstly, Choi and Eastman^1^ reviewed the thermophysical features of nanoparticles. Numerous scholars have written significant reports on the thermal behaviour of nanoparticles and nanoliquids. Ali et al.^2^ conducted a thorough analysis of the effects of ohmic heating on the flow of nanofluids. Waqas et al.^3^ reviewed the Maxwell nanoliquid stream initiated by a cylinder by considering bioconvection. Khan et al.^4^ studied the flow of nanoliquid with magnetic effect and activation energy. Zhou et al.^5^ scrutinized the Williamson nanofluid stream on taking account of bioconvection and double diffusion effects. See^6–10^ for more recent studies on nanofluid. Recently, hybrid nanofluid has gained more attention from researchers. This is due to its higher thermal conductivity compared to the nanofluids; and thus the hybrid nanofluid serves as a better choice for heat transfer in thermal devices or systems^11–20^. A hybrid nanofluid is an engineered suspension of two separate solid nanoparticles amalgamated in a base liquid. Its thermal conductivity is higher than that of a simple nanofluid. Anuar et al.^21^ explored magnetohydrodynamic flow of copper-alumina based hybrid nanoliquid and found that the boundary layer separation is delayed by an increasing magnetic field. The study also shows their two solutions; stable solution and unstable solution. Mabood et al.^22^ typified the effect of heat radiation on a hybrid nanofluid’s MHD flow and the results show that flow velocity declines as mass concentration increases. Gowda et al.^23^ examined a fluid stream containing dual nanoparticles over a rotating disk on accounting particle deposition. The upward speed of the disk movement led to an increase in both the tangential and radial velocity. Mass transfer also declines as the thermophoresis increases.

Many contemporary heat exchange systems that require very high temperatures rely on thermal radiation in flow and heat transfer operations. Thermal radiation is a kind of heat transfer phenomenon that distributes warmth energy via liquid particles. The stimulation of the radiation impact on the magnetohydrodynamic stream has a huge appeal in a plethora of industrial and technical operations involving high temperatures, such as the manufacturing of petroleum pumps, the production of electric chips, paper plates, and the cooling of metallic components. Khan et al.^24^ investigated the thermophoresis effects on the second-grade liquid flow with radiation effect over an expanding surface. The equations were rendered dimensionless and the resulting non-linear ordinary differential equation was solved using the Homotopy Analysis Method. By increasing the film thickness and magnetic field strength, it was discovered that velocity profiles are reduced significantly. The temperature profiles rises with an increase in the thermal conductivity parameter. In a study by Animasaun et al.^25^, it was discovered that the Nusselt number $-\theta '\left( 0\right)$ increases with Prandtl number at an optimal rate of 1.53 when the transmission of heat energy through electro-magnetics waves is minimal.

The flow and heat passage of different liquids via a MF is widely used in a variety of industrial applications and technology. Due to various uses in engineering processes and energy extraction, liquid flow over a stretched surface with MFs has attracted considerable interest. Numerous researches on MHD flow on a stretching sheet/plate have been published during the last several decades. Irfan et al.^26^ swotted the Maxwell nanoliquid flow through a magnetically fielded cylinder. The magnetic field strength enhanced the temperature and concentration profiles while inhibiting the velocity profile of Maxwell nanofluid. The melting and magnetic effects on the Casson liquid flow were shown by Nandeppanavar et al.^27^. The study explored the heat transfer and concentration of double-diffusive free convection flow of electrically-conducting Casson fluid towards a stagnation-point. The velocity profiles are found to reduce as the magnetic field strength increased. The outcomes of applying MF on the chemically reacting Casson nanoliquid was typified by Kumar et al.^28^. Magnetic field was found to also inhibit the flow velocity. Khan et al.^29^ and Oyem et al.^30^ swotted the impact of MF on a dissipative stream of nanoliquid under Robin condition. Stretching of models are important due to their many applications in manufacturing, such as, boundary layer along the liquid film concentration process and polymer sheet extrusion from substrate. See^9,31^ for more studies on MHD flow of different fluids.

Coriolis force is the force responsible for the deflection in the direction of a flowing fluid. In the fundamental flow equations, the Coriolis force is as important as any other inertial forces, magnetohydrodynamic forces, and viscous forces. The pressure gradient force, gravitational force, centrifugal force and frictional force all act on any liquid flow on the surface of the earth. The opposite is the case for the atmosphere and water, where Coriolis force has no significant influence on all transport phenomena. When the liquid motion speed is small in comparison to the rotation speed, the Coriolis effect becomes negligible, which is why the Coriolis effect is not readily experienced on earth. From past decades, numerous researchers are exploring the Coriolis force impact on diverse liquid streams^32–38^ and in each of the studies, Coriolis effect was found to be significant on the flow velocity.

It is important to note that based on the available research literature, no research has been done on the simultaneous impact of the heat radiation and Coriolis force on the water transporting copper and alumina nanoparticles on a rotating exponentially stretching plate. Hence, this study is novel and has practical significance in mathematics and engineering, and will open a space for further research. The following research questions are answered in this study;

1. How does increasing Coriolis effect impact the flow of copper-alumina-water-based hybrid nanofluid subjected to thermal radiation?
2. How does raising the the size of the MF strength affect the skin friction and heat transfer rate coefficients in the flow of copper-alumina-water-based hybrid nanofluid flow
3. How does increasing inclination angle affect the flow of water-based hybrid nanofluid?
4. How does increasing volume fraction affect heat transfer rate in the flow of water-based hybrid nanofluid?

## Governing equations and methodology

**Figure 1:**
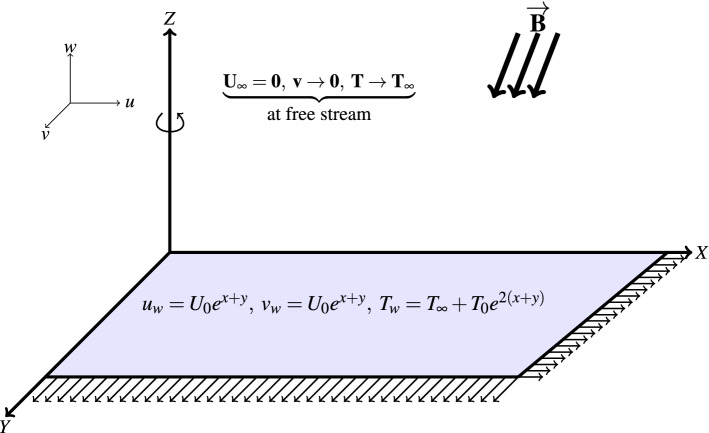
Flow configuration.

This study analyses a 3D boundary layer flow of electrically conducting water-based hybrid nanofluid past an exponentially stretched sheet. Figure 1 shows the set-up of flow configuration. The sheet rotates at an angular velocity $\Omega$ and the flow is steady, laminar and incompressible. An inclined MF of strength *B* is applied to the surface at an angle $\gamma .$ Following the formulations of Nayak et al.^39^ and Oke et al.^35^, the equations governing the flow is given in Eqs. (1–4);

1 $$\begin{aligned}&u_{x}+v_{y}+w_{z}=0, \end{aligned}$$

2 $$\begin{aligned}{}&uu_{x}+vu_{y}+wu_{z}=\frac{\mu _{hnf}}{\rho _{hnf}}u_{zz}+g^{*}\beta \left( T-T_{\infty }\right) -\frac{\sigma _{hnf}B_{0}^{2}u}{\rho _{hnf}}\sin \gamma +2\Omega v\end{aligned}$$

3 $$\begin{aligned}{}&uv_{x}+vv_{y}+wv_{z}=\frac{\mu _{hnf}}{\rho _{hnf}}\frac{\partial ^{2}v}{\partial z^{2}}+g^{*}\beta \left( T-T_{\infty }\right) +\frac{\sigma _{nf}B_{0}^{2}v}{\rho _{hnf}}\sin \gamma -2\Omega u\end{aligned}$$

4 $$\begin{aligned}{}&uT_{x}+vT_{y}+wT_{z}=\left( \alpha _{hnf}+\frac{16\sigma ^{*}T_{\infty }^{3}}{3k^{*}\left( \rho c_{p}\right) _{hnf}}\right) T_{zz}, \end{aligned}$$

The boundary and initial conditions are given in Eqs. (5) and (6);

5 $$\begin{aligned}{}&u=U_{w}=U_{0}e^{x+y},\;v=V_{w}=U_{0}e^{x+y},w=0,\;T=T_{w}=T_{\infty }+T_{0}e^{2\left( x+y\right) }\;\text{ at } z=0, \end{aligned}$$

6 $$\begin{aligned}{}&u\rightarrow 0,\;v\rightarrow 0,\;T\rightarrow T_{\infty },\;\text{ as }\;z\rightarrow \infty . \end{aligned}$$

The effective dynamic viscosity $\mu _{hnf}$ and effective density $\rho _{hnf}$ of the hybrid nanofluid^21^ are defined in Eq. (7) below;

7 $$\begin{aligned} \mu _{hnf}=\frac{\mu _{bf}}{\sqrt{\left( 1-\phi \right) ^{5}}},\quad \rho _{hnf}=\left( 1-\phi \right) \rho _{bf}+\phi _{1}\rho _{1}+\phi _{2}\rho _{2}, \end{aligned}$$

where $\phi$ is the overall volume fraction defined as $\phi =\phi _{1}+\phi _{2}$^10^. The effective thermal diffusivity $\alpha _{hnf}$ and the specific heat capacity $\left( \rho c_{p}\right) _{hnf}$^40–42^ are defined as shown in Eqs. (8) and (9);

8 $$\begin{aligned}{}&\alpha _{hnf}=\frac{k_{hnf}}{\left( \rho c_{p}\right) _{hnf}},\quad \frac{\left( \rho c_{p}\right) _{hnf}}{\left( \rho c_{p}\right) _{bf}}=1-\phi +\phi _{1}\frac{\left( \rho c_{p}\right) _{1}}{\left( \rho c_{p}\right) _{bf}}+\frac{\phi _{2}\left( \rho c_{p}\right) _{2}}{\left( \rho c_{p}\right) _{bf}}, \end{aligned}$$

9 $$\begin{aligned}{}&\frac{k_{hnf}}{k_{bf}}=\dfrac{\left( 1+2\phi \right) \left( \phi _{1}k_{1}+\phi _{2}k_{2}\right) +2\left( 1-\phi \right) \phi k_{bf}}{\left( 1-\phi \right) \left( \phi _{1}k_{1}+\phi _{2}k_{2}\right) +\left( 2+\phi \right) \phi k_{bf}}. \end{aligned}$$

The skin friction and heat transfer rate along the *x*- and *z*-axes are the quantities of engineering relevance, and they are given in Eq. (10) as

10 $$\begin{aligned} Cf_{x}=\frac{\tau _{x}}{\rho _{hnf}U_{w}^{2}},\quad Cf_{z}=\frac{\tau _{y}}{\rho _{hnf}U_{w}^{2}},\quad \text{ and } \quad Nu=\frac{zq_{w}}{\kappa _{hnf}\left( T_{w}-T_{\infty }\right) }. \end{aligned}$$

respectively. The shear stress $\tau$ along the *x*- and *y*- directions on the wall and the wall heat flux $q_{w}$ are defined as the following quantities evaluated at the wall $\left( \text{ i.e. } z=0\right) ;$

$$\begin{aligned} \tau _{x}=\mu _{hnf}\frac{\partial u}{\partial z},\;\tau _{z}=\mu _{hnf}\frac{\partial v}{\partial z},\;\text{ and } \;q_{w}=-\kappa _{hnf}\frac{\partial T}{\partial z}. \end{aligned}$$

### Methodology

The partial differential equations (1–4) with the initial and boundary conditions (5 and 6) are nondimensionalised using the similarity variables given in Eqs. (11–13) below;

11 $$\begin{aligned}{}&\eta =\left( \frac{U_{0}}{2\nu _{bf}}\right) ^{\frac{1}{2}}z\exp \left( \frac{x+y}{2}\right) ,u=U_{0}f'\left( \eta \right) \exp \left( x+y\right) , \end{aligned}$$

12 $$\begin{aligned}{}&v=U_{0}g'\left( \eta \right) \exp \left( x+y\right) ,\;T=T_{\infty }+\theta T_{0}\exp \left( 2x+2y\right) ,\end{aligned}$$

13 $$\begin{aligned}{}&w=-\left( \frac{\nu _{bf}U_{0}}{2}\right) ^{\frac{1}{2}}\left( f+\eta f'+g+\eta g'\right) \exp \left( \frac{x+y}{2}\right) , \end{aligned}$$

and the resulting dimensionless equations are shown in Eqs. (14)–(16) below

14 $$\begin{aligned}{}&A_{1}f'''+f''\left( f+g\right) +Kg'+2Gr\Theta -2Mf'\sin \gamma -2f'\left( f'+g'\right) =0\end{aligned}$$

15 $$\begin{aligned}{}&A_{1}g'''+g''\left( f+g\right) -Kf'+2Gr\Theta -2Mg'\sin \gamma -2g'\left( f'+g'\right) =0\end{aligned}$$

16 $$\begin{aligned}{}&\left( 1+\frac{4}{3}R\right) A_{2}\Theta ''+Pr\left( f+g\right) \Theta '-4Pr\Theta \left( f'+g'\right) =0 \end{aligned}$$

with

17 $$\begin{aligned}{}&f=-g,\;f'=1,\;g'=1,\;\Theta =1,\quad \text{ at } \eta =0\end{aligned}$$

18 $$\begin{aligned}{}&f'\rightarrow 0,\;g'\rightarrow 0,\;\Theta \rightarrow 0,\quad \text{ as } \eta \rightarrow \infty . \end{aligned}$$

where the dimensionless parameters are given in Eqs. (19)–(21) below;

19 $$\begin{aligned}{}&Gr=\frac{g\beta T_{0}}{U_{0}^{2}},\;M=\frac{\sigma _{hnf}B_{0}^{2}}{\rho _{hnf}U_{0}},\;Pr=\frac{\nu _{bf}}{\alpha _{bf}},\;R=\frac{4\sigma ^{*}T_{\infty }^{3}}{\alpha _{hnf}k^{*}\left( \rho c_{p}\right) _{hnf}},\;K=\frac{4\Omega }{U_{0}e^{x+y}},\end{aligned}$$

20 $$\begin{aligned}{}&A_{2}=\dfrac{\left( 1+2\phi \right) \left( \phi _{1}k_{1}+\phi _{2}k_{2}\right) +2\left( 1-\phi \right) \phi k_{bf}}{\left( 1-\phi \right) \left( \phi _{1}k_{1}+\phi _{2}k_{2}\right) +\left( 2+\phi \right) \phi k_{bf}}\left( 1-\phi +\phi _{1}\frac{\left( \rho c_{p}\right) _{1}}{\left( \rho c_{p}\right) _{bf}}+\phi _{2}\frac{\left( \rho c_{p}\right) _{2}}{\left( \rho c_{p}\right) _{bf}}\right) ^{-1},\end{aligned}$$

21 $$\begin{aligned}{}&A_{1}=\left( 1-\phi +\frac{\phi _{1}\rho _{1}}{\rho _{bf}}+\frac{\phi _{2}\rho _{2}}{\rho _{bf}}\right) ^{-1}\left( 1-\phi \right) ^{-2.5}. \end{aligned}$$

The system of Eqs. (14–16) with the boundary conditions are reformulated by setting

$$\begin{aligned}{}&H_{1}=f,H_{2}=f',H_{3}=f'',H_{4}=g,\\&H_{5}=g',H_{6}=g'',H_{7}=\Theta ,H_{8}=\Theta ' \end{aligned}$$

to give

22 $$\begin{aligned} \frac{d}{d\eta }H_{1}=&H_{2},\quad \frac{d}{d\eta }H_{2}=H_{3},\end{aligned}$$

23 $$\begin{aligned} \frac{d}{d\eta }H_{3}=&-\frac{1}{A_{1}}\left( KH_{5}+2GrH_{7}-2MH_{2}\sin \gamma +H_{3}\left( H_{1}+H_{4}\right) -2H_{2}\left( H_{2}+H_{5}\right) \right) ,\end{aligned}$$

24 $$\begin{aligned} \frac{d}{d\eta }H_{4}=&H_{5},\quad \frac{d}{d\eta }H_{5}=H_{6},\end{aligned}$$

25 $$\begin{aligned} \frac{d}{d\eta }H_{6}=&-\frac{1}{A_{1}}\left( -KH_{2}+2GrH_{7}-2MH_{5}\sin \gamma +H_{6}\left( H_{1}+H_{4}\right) -2H_{5}\left( H_{2}+H_{5}\right) \right) ,\end{aligned}$$

26 $$\begin{aligned} \frac{d}{d\eta }H_{7}=&H_{8},\quad \frac{d}{d\eta }H_{8}=-\left( \left( 1+\frac{4}{3}R\right) A_{2}\right) ^{-1}\left( Pr\left( H_{1}+H_{4}\right) H_{8}-4PrH_{7}\left( H_{2}+H_{5}\right) \right) , \end{aligned}$$

with the initial conditions at $\eta =0;$

27 $$\begin{aligned} H_{1}=f_{w},\,H_{2}=1,\,H_{3}=s_{1},\,H_{4}=-f_{w},\,H_{5}=1,\,H_{6}=s_{2},\,H_{7}=1,\,H_{8}=s_{3}, \end{aligned}$$

and $s_{1},s_{2},s_{3}$ are chosen to satisfy the boundary conditions at $\eta _{\infty }$;

$$\begin{aligned} H_{2}=0,\,H_{5}=0,\,H_{7}=0. \end{aligned}$$

This coupled system of ordinary differential equations is highly non-linear and cannot be solved analytically. The semi-analytical method of solutions can be used but they require a lot of computation time^43^. Hence, the numerical methods provide a more efficient and computationally-economical approach to finding the solutions. The solution of Eqs. (22)–(26) with Eq. (27) are found numerically by using the bvp4c function in MATLAB with the absolute and the relative tolerance of $10^{-8}$^43,44^. The results of this investigation were verified by comparing them to the bvp5c results and shown in Table 1.

**Table 1:** validation of results for $Pr=6.2$.

$\phi$	*M*	*R*	$\gamma$	*K*	$f''\left( 0\right)$		$-\Theta '\left( 0\right)$	
					bvp4c	bvp5c	bvp4c	bvp5c
0.012	5.00	2.00	$30^{\circ }$	0.100	−2.614848	−2.614848	29.375192	29.375192
0.050	1.00	2.00	$30^{\circ }$	0.100	−1.858095	−1.858094	37.670152	37.670152
0.050	5.00	0.50	$30^{\circ }$	0.100	−2.819812	−2.819812	75.602883	75.602882
0.050	5.00	2.00	$10^{\circ }$	0.100	−2.037720	−2.037721	37.625935	37.625934
0.050	5.00	2.00	$30^{\circ }$	0.001	−2.719729	−2.719730	37.461254	37.461254

## Discussion of results

The outcomes associated with the heat transfer in water-based hybrid nanofluid flow along a rotating and exponentially-stretching plate are hereby discussed. The governing equations are modelled with the presence of Coriolis force and MF. The study elucidates the significance of involved controlling numerous somatic factors in the modelling equations using graphs and tables. Variation in drag coefficient $f''\left( 0\right)$ and $g''\left( 0\right)$ and the Nusselt number $-\Theta '\left( 0\right)$ for varying pertinent parameters are tabulated in Table 2.

Practically, increase in *M* and *K* are consequences of increased magnetic field strength and surface rotation respectively. The presence of magnetic field around the electrically-conducting fluid tends to oppose flow while rotation propels the flow forward in the direction of the flow. Raising the values of *M* and *K* improves $f'\left( 0\right)$ but a conflicting trend is seen for growing values of volume fraction, *R* and $\gamma .$ Further, an upsurge in nanoparticle volume fraction $\phi$ increases the rate of heat transfer $-\Theta '\left( 0\right)$ but a conflict trend is seen for growing values of *M*,  *K*,  *R* and $\gamma .$ By raising the volume fraction of the nanoparticles, the thermal conductivity of the nanofluid is improved and therefore the thickness of the thermal boundary layer grows, resulting in an increasing rate of heat transfer. The rate of thermal heat transfer is substantially influenced by thermal radiation. When the volume of nanoparticles grows, the heat transfer rate falls as thermal radiation rises.

**Figure 2:**
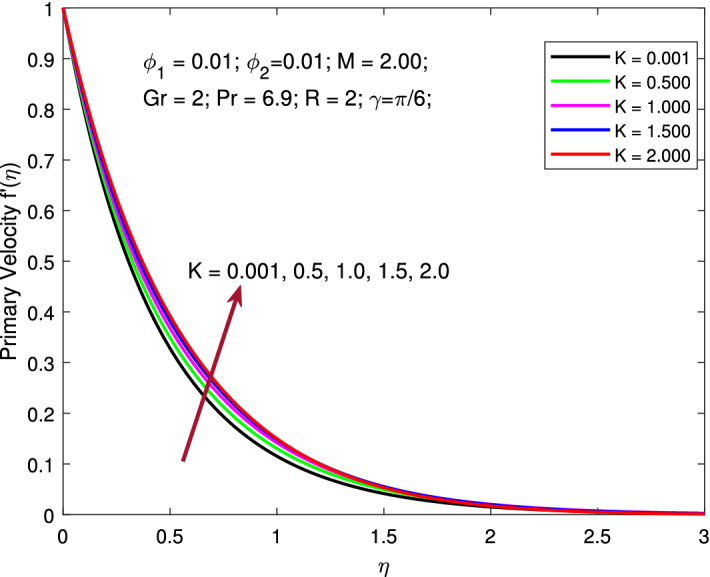
Variation of primary velocity with Coriolis force.

**Figure 3:**
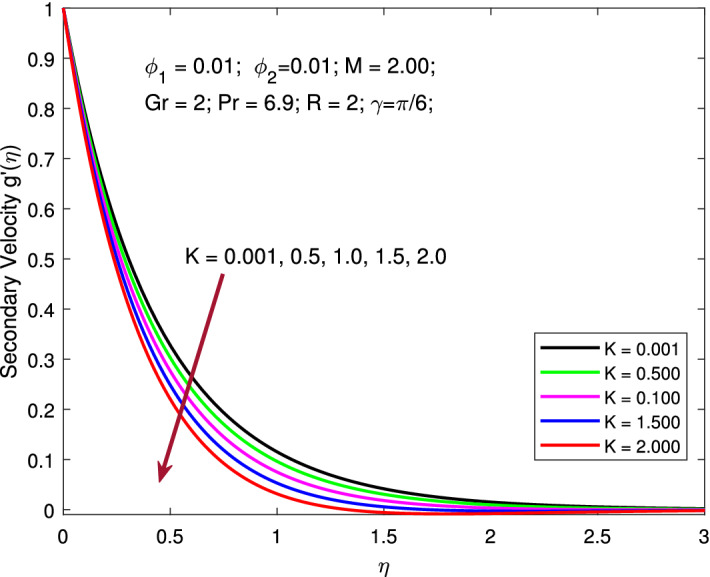
Variation of secondary velocity with Coriolis force.

Figures 2 and 3 are designed to explore the role of rotation parameter *K* on primary and secondary velocity profiles. Here, to obtain the variation of the pertinent profiles the parameters are kept fixed as $M=2,$ $\phi _{1}=\phi _{2}=0.01,$ $Gr=2,$ $Pr=6.9,$ $R=2,$ and $\gamma =\pi /6,$ while the values of rotation parameter $K=0.001,0.5,1,1.5,2$ is varied. The Coriolis force becomes stronger by increasing the values of *K*,  which leads to an upsurge in the primary velocity profile. Further, increasing rotation parameter *K* causes a decline in the secondary velocity profile. This is all because of the significant influence of Coriolis force along with the stretching influence. The inertia force accountable for the deviation of the trajectory of liquid flow along a spinning surface is known as the Coriolis force and it becomes stronger by raising *K*,  which leads to upsurge in primary velocity profile. Further, larger value of rotation parameter *K* reduces the secondary velocity profile. Physically, when *K* becomes larger, the rotation effects take precedence over the stretching effects, slowing the flow velocity. This is all because of the significant influence of Coriolis force along with the stretching influence.

**Figure 4:**
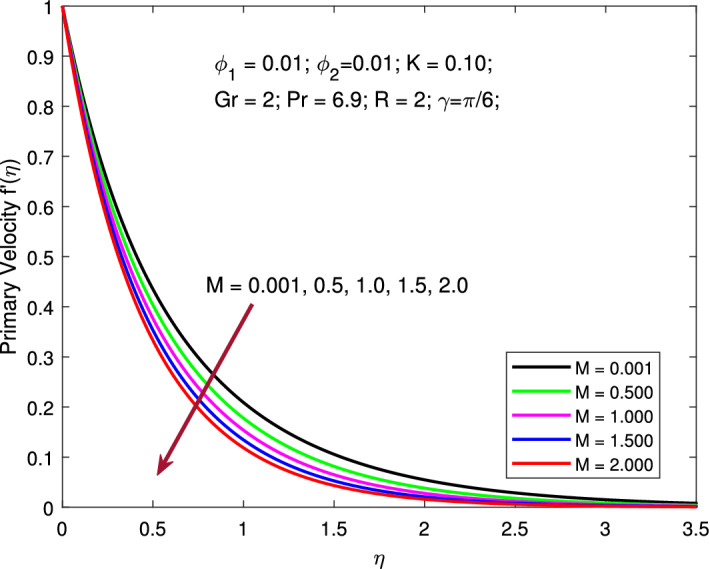
Variation of primary velocity with MF strength.

**Figure 5:**
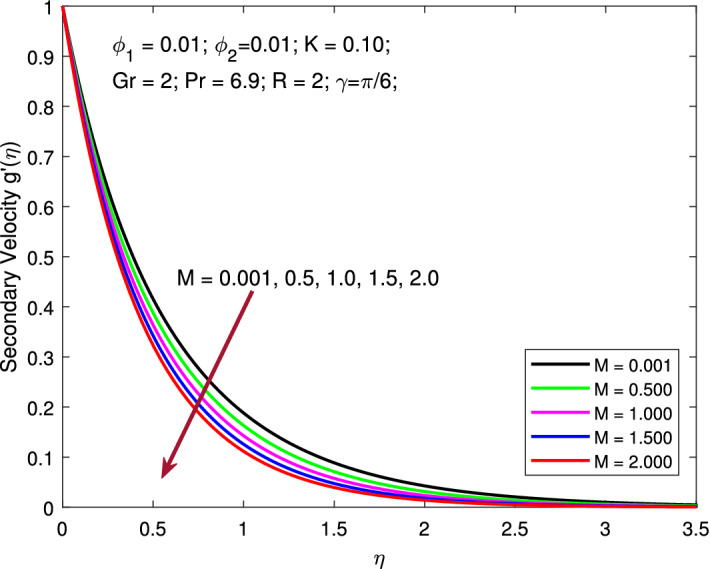
Variation of secondary velocity with MF strength.

Figures  4 and 5 elucidate the leverage of *M* on both primary and secondary velocity profiles. All parameters are kept fixed as $\phi _{1}=\phi _{2}=0.01,$ $K=0.1,$ $Gr=2,$ $Pr=6.9,$ $R=2,$ and $\gamma =\pi /6$ while magnetic parameter $M=0.001,0.5,1,1.5,2$ is varied to examine its consequence on the flow fields. Increase in *M* inhibits the flow and thereby causes a reduction in the velocity profiles. The presence of an MF in the flow region has been shown to slow down flow velocity. The magnetic force adds a layer of resistance to the flow and slows down the flow. The existence of a transverse MF induces the Lorentz force, which acts as a retarding force on the velocity field of base liquid and nanoparticles. As a result, as seen in the figures, this negative body force slows the boundary layer flow and inhibits momentum diffusion.

**Figure 6:**
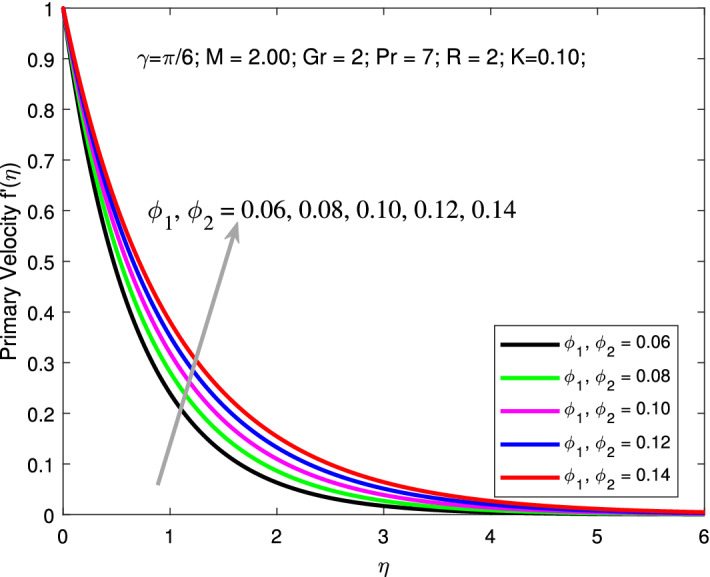
Primary velocity with $\phi$.

**Figure 7:**
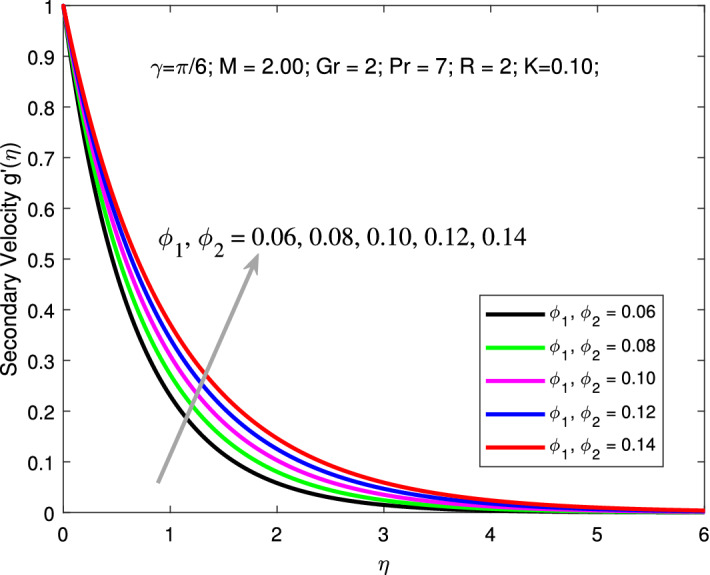
Variation of secondary velocity with nanoparticle volume fraction.

**Figure 8:**
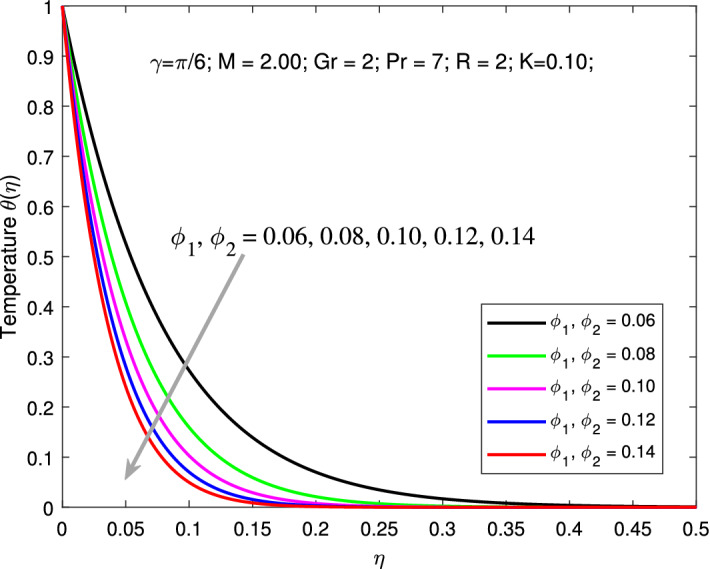
Variation of temperature with nanoparticle volume fraction.

**Figure 9:**
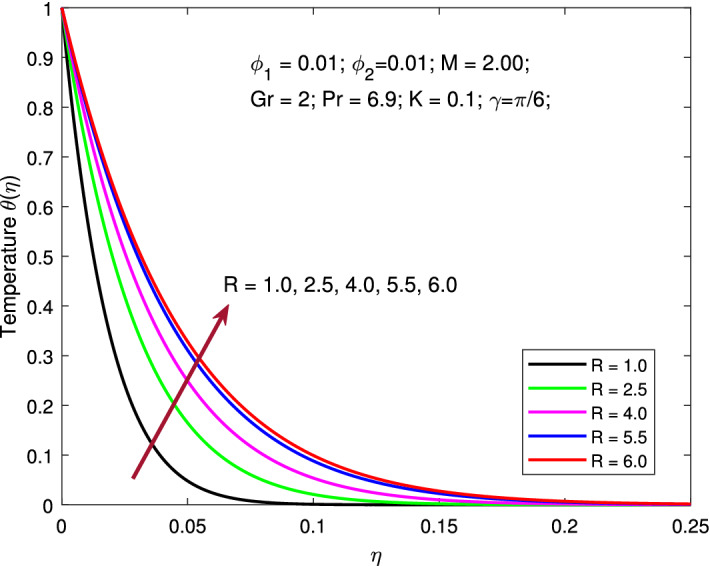
Variation of temperature with thermal radiation.

**Figure 10:**
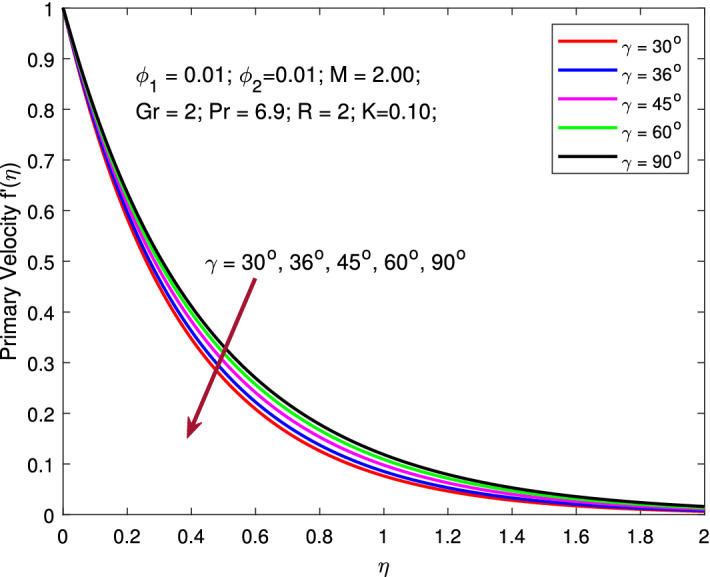
Variation of temperature with MF inclination angle.

**Figure 11:**
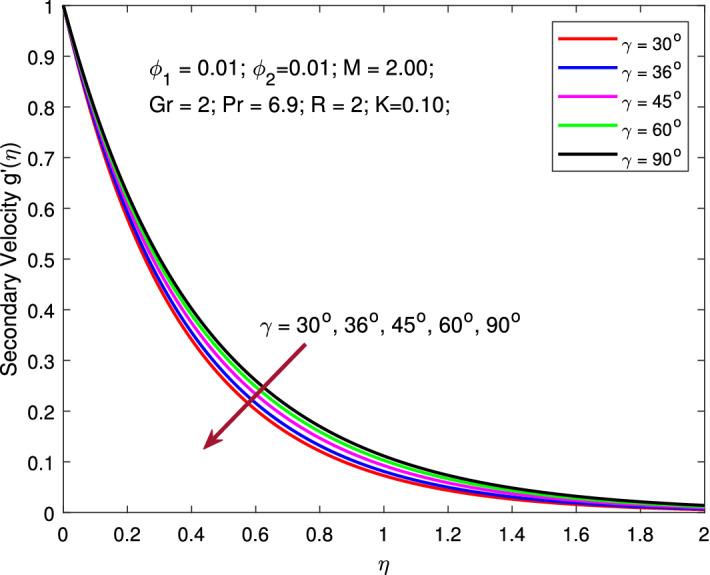
Variation of temperature with MF inclination angle.

Variation of primary and secondary velocities and thermal profiles for various values of $\phi _{1}$ and $\phi _{2}$ are shown in Figs. 6, 7 and 8 . An increase in values of $\phi _{1}$ and $\phi _{2}$ boosts the primary and secondary velocities but declines the thermal profiles. Here, the parameters are kept fixed as $M=2,$ $K=0.1,$ $Gr=2,$ $Pr=6.9,$ $R=2,$ and $\gamma =\pi /6$ while the values of nanoparticle volume fraction $\phi _{1}=\phi _{2}$ is varied between 0.01 to 0.005. Increasing solid volume fractions enhances the thickness of the boundary layer. As a result, the fluid will flow faster which increases primary, and secondary velocity profile. Addition of solid nanoparticles to the base fluid will gradually decline the thermal distribution due to decrease in the thickness of the related boundary layer. Figures 9 is depicted to elucidate the influence of *R* on thermal profile. Here, the parameters are kept fixed as $M=2,$ $K=0.1,$ $Gr=2,$ $Pr=6.9,$ $\phi _{1}=\phi _{2}=0.01,$ and $\gamma =\pi /6$ while, the values of *R* is varied between 1 to 6. The effect of thermal radiation increases the temperature profile, as seen in this figure. Radiative heat transmission is less effective than conductive heat transport, lowering the buoyancy force. High *R* delivers more heat to functional nanofluids, which indicates an increment in thermal profile. The variation is more gradual than when the radiation parameter is at a lower value. When the radiation parameter is set to a higher value, the fluid is heated more and more, increasing the thermal profile. Figures 10 and 11 signify the change in the pattern of the primary and secondary flow velocities for increase in values of angle of inclination of MF. Here, the parameters are kept fixed as $M=2,$ $K=0.1,$ $Gr=2,$ $Pr=6.9,$ $\phi _{1}=\phi _{2}=0.01$ and $R=2$ while, the values of nanoparticle volume fraction $\gamma$ is varied between $30^{\circ }$ to $90^{\circ }$. Primary and secondary flow velocity decreases as the angle of inclination of MF in the region inclines. Increased angle of inclination of MF improves molecular movements and interactions, resulting in increased viscous force. When the angular velocity is increased, the average kinetic energy is predicted to grow as well. This gradually causes the fluid velocity to decline.

**Table 2:** Skin frictions and local Nusselt number for various values of $\phi ,M,R,\gamma ,K$ while $Pr=6.2$.

$\phi$	*M*	*R*	$\gamma$	*K*	$f''\left( 0\right)$	$g''\left( 0\right)$	$-\Theta '\left( 0\right)$
0.012	5.00	2.00	$30^{\circ }$	0.100	−2.614848	−2.653190	29.375192
0.022					−2.716640	−2.755342	39.176489
0.030					−2.769340	−2.808350	45.232950
0.040					−2.819908	−2.859284	51.504346
0.050					−2.860428	−2.900138	56.787260
0.050	1.00				−1.858095	−1.917526	37.670152
	2.00				−2.098457	−2.149596	37.610940
	1.50				−1.981728	−2.036512	37.639769
	2.00				−2.098457	−2.149596	37.610940
	2.50				−2.209400	−2.257567	37.583620
	5.00	0.50			−2.819812	−2.858306	75.602883
		1.75			−2.715837	−2.754440	40.093889
		3.00			−2.648445	−2.687156	30.466647
		4.25			−2.595166	−2.633984	25.495421
		5.50			−2.550017	−2.588941	22.336609
		2.00	$10^{\circ }$		−2.037720	−2.090680	37.625935
			$20^{\circ }$		−2.401085	−2.444954	37.535913
			$36^{\circ }$		−2.854502	−2.890931	37.422844
			$45^{\circ }$		−3.052005	−3.085977	37.373380
			$90^{\circ }$		−3.492588	−3.522169	37.261920
			$30^{\circ }$	0.001	−2.719729	−2.720115	37.461254
				0.150	−2.691103	−2.749039	37.461214
				0.300	−2.662593	−2.778443	37.461093
				0.450	−2.634400	−2.808122	37.460891
				0.600	−2.606534	−2.838065	37.460617

## Conclusion

The heat transference in water-based hybrid nanofluid flow over a rotating exponentially stretching plate is explored in this analysis. The governing equations are modelled in the occurrence of Coriolis force and MF. A reformulation of the governing equations in their dimensionless forms using similarity transformation is first carried out and the resulting equations are solved using the finite difference scheme. The study elucidates the significances of involved controlling numerous somatic factors in the modelling equations with the graphs and tables. The most important outcomes of this study are:

- The rise in rotation parameter results in stronger Coriolis force, which leads to upsurge in primary velocity profile but declines the secondary velocity profile.
- Increase in MF parameter declines the flow in both velocity profiles due to the presence of a transverse MF that generates the Lorentz force, which acts as a inhibiting force on the velocity field.
- The rise in the MF inclination angle in the region improves molecular movements and interactions, resulting in increased viscous force as a result primary and secondary flow velocity decreases.
- The escalation in values of radiation parameter delivers more heat to functional nanofluids which augments the heat transfer.
- Increasing MF strength and rotation parameters improves the skin friction coefficient conflict trend is seen for growing values of volume fraction, radiation parameter and MF inclination angle.
- The upsurge in values of volume fraction improves the heat transfer rate but the opposite is seen for growing values of MF inclination angle, MF strength, rotation and radiation parameters.

## Data Availability

All data generated or analysed during this study are included in this published article

## List of symbols

$B_{0}$: Magnetic field strength $\left( \mathrm{A\, L}^{-1}\right)$
$c_{p}$: Specific heat capacity $\left( \mathrm{J\;kg}^{-1}\, \mathrm{K}^{-1}\right)$
$g^{*}$: Acceleration due to gravity $\left( \mathrm{L \,T}^{-2}\right)$
*Gr*: Grashof number
*k*: Thermal conductivity $$\left( \mathrm{M\,L\,T}^{-3}\, \mathrm{K}^{-1}\right)$$
$k^{*}$: Mean absorption coefficient $\left( \mathrm{L}^{-1}\right)$
*K*: Rotation parameter
*M*: Magnetic field parameter
MF: Magnetic field
*Pr*: Prandtl number
*R*: Thermal radiation parameter
*T*: Dimensional fluid temperature $\left( \mathrm{K}\right)$
*x*, *y*, *z*: Cartesian coordinates 3D space $\left( \mathrm L\right)$
*u*, *v*, *w*: Velocity component in the $x,y,z\text {-}$directions$\left( \mathrm{L\,T}^{-1}\right)$

## Greek symbols

$$\nu$$: Kinematic viscosity $$\left( \mathrm{L}^{2}\,\mathrm{T}^{-1}\right)$$
$\sigma ^{*}$: Stefan–Boltzmann constant $\left( \mathrm{W}\;\mathrm{L}^{-2} \mathrm{K}^{-4}\right)$
$$\sigma$$: Electrical conductivity $$\left( \mathrm{M}^{-1} \,\mathrm{L}^{-3} \mathrm{T}^{3}A^{2}\right)$$
$$\alpha$$: Thermal diffusivity $$\left( \mathrm{L}^{2}\, \mathrm{T}^{-1}\right)$$
$$\rho$$: Density $$\left( \mathrm{M}\, \mathrm{L}^{-3}\right)$$
$$\mu$$: Viscosity $$\left( \mathrm{M}\, \mathrm{L}^{-1}\, \mathrm{T}^{-1}\right)$$
$\Omega$: Angular velocity of the surface $\left( \mathrm{T}^{-1}\right)$
$\gamma$: Angle of inclination of magnetic field
$\phi$: Overall nanoparticle volume fraction
$$\phi _{i},i=1,2$$: Volume fraction
$\beta$: Coefficient of thermal expansion $\left( \mathrm{K}^{-1}\right)$

## Subscripts

*bf*: Base fluid
*hnf*: Hybrid nanofluid
*w*: Surface wall
1: Cu nanoparticle
2: Al$$_{2}\mathrm{O}_{3}$$ nanoparticle
$$\infty$$: Free stream
